# Acceptance and Commitment Therapy in a Low-Income Country in Sub-Saharan Africa: A Call for Further Research

**DOI:** 10.3389/fpubh.2021.732800

**Published:** 2021-09-23

**Authors:** Yonas E. Geda, Janina Krell-Roesch, Yaphet Fisseha, Aida Tefera, Teferra Beyero, Deborah Rosenbaum, Thomas G. Szabo, Mesfin Araya, Steven C. Hayes

**Affiliations:** ^1^Department of Neurology, Barrow Neurological Institute, Phoenix, AZ, United States; ^2^Department of Health Sciences Research, Mayo Clinic, Rochester, MN, United States; ^3^Institute of Sports and Sports Science, Karlsruhe Institute of Technology, Karlsruhe, Germany; ^4^Clinical PsyD Department, The Chicago School of Professional Psychology, Washington, DC, United States; ^5^Department of Psychiatry, Eka Kotebe Hospital, Addis Ababa, Ethiopia; ^6^Needham Psychotherapy Associates, Needham, MA, United States; ^7^School of Behavior Analysis, Florida Institute of Technology, Melbourne, FL, United States; ^8^Department of Psychiatry, College of Health Sciences, School of Medicine, Addis Ababa University, Addis Ababa, Ethiopia; ^9^Department of Psychology, University of Nevada, Reno, NV, United States

**Keywords:** Acceptance and Commitment Therapy, psychological flexibility, health extension workers, sub-Saharan Africa, Ethiopia, Cognitive Behavioral Therapy

## Abstract

A worsening trend of critical shortages in senior health care workers across low- and middle-income countries (LMICs) in sub-Saharan Africa has been documented for decades. This is especially the case in Ethiopia that has severe shortage of mental health professionals. Consistent with the WHO recommended approach of task sharing for mental health care in LMICs, Acceptance and Commitment Therapy (ACT), which is an empirically validated psychological intervention aimed at increasing psychological flexibility, may be delivered by trained laypersons who have a grassroots presence. In this paper, we discuss the need for and potential role of ACT to be delivered by health extension workers (HEWs) to address mental health care needs across Ethiopia. To this end, we also reviewed previous studies that have examined the effectiveness of ACT-based interventions in African countries including in Nigeria, Sierra Leone, Uganda, and South Africa. All studies revealed significant improvements of various mental health-related outcome measures such as decreased psychological distress and depressive symptoms, or increased subjective wellbeing and life satisfaction in the groups that received an ACT-based intervention. However, to date, there is no study that applied ACT in Ethiopia. Thus, more research is warranted to examine the effectiveness and, if proven successful, to scale up a task sharing approach of an ACT-based intervention being delivered by trained HEWs at a grassroots level, possibly paving the way for an innovative, sustainable mental health service in Ethiopia as well as other African LMICs.

## Introduction

Sustainable health care requires a combination of infrastructure, human resources, and supply chain management. When any one of these three factors are limited, the result is a degradation of available services. Sub-Saharan Africa has faced ongoing critical shortages of health care workers for decades (1, 2), and migration of professional health care workers across English-speaking African nations shows a consistent pattern of movement from nations with weaker to more developed healthcare infrastructures (3). Thus, infrastructural and supply chain administration enhancements have not yet led to improved health care outcomes. The World Health Organization (WHO) estimates that 78% of all sub-Saharan African nations suffered from critical health care worker shortages with substantial impacts upon child and adult mortality, susceptibility to disease and infection, and mental health outcomes (4). Of these, the variable with the most substantial impact upon public health is mental health. That is, when people connect with their values and act accordingly, the probability of their following health guidelines for disease and infection prevention, getting checkups, vaccinations, healthy food, sleep, and physical exercise increases (5).

The time-honored cornerstone of mental health intervention is anchored upon the biopsychosocial model (6). A full-blown mental illness is considered a downstream event. Ideally, society would like to promote mental health and prevent mental illness. In the Western world, there are wide-spread and expensive pharmacological interventions to manage mental illnesses. However, such expensive medications are not sustainable in sub-Saharan Africa. Therefore, a scientifically sound, empirically validated psychological intervention that can be delivered by paraprofessionals at a grassroots level may bring about a paradigm shift on using a less expensive, and perhaps in the long-term, more sustainable mental health intervention.

Ideally, such an intervention should be effective for the prevention of mental illness as well as treatment of an existing mental illness. One such possible intervention that may meet the aforementioned premises is Acceptance and Commitment Therapy (ACT), which is an empirically validated psychological intervention that has its roots in the school of Cognitive Behavioral Therapy (CBT). There are a few reasons as to why we selected ACT for the theme of this manuscript: (1) Laypersons can be trained to deliver ACT under the supervision of mental health professionals. (2) ACT leaders have taken deliberate steps to ensure that ACT is not the exclusive domain of mental health professionals, thus avoiding the need for board certification or similar red tapes that could have hampered supervised training of laypersons from delivering ACT. (3) The customary approach of psychiatric interventions is to “delete” or decrease psychiatric signs and symptoms. However, ACT's primary goal is not to eliminate psychiatric symptoms, but rather to cultivate psychological skill sets, including willingness to make room for uncomfortable emotions while pursuing personally meaningful values. (4) The transdiagnostic approach of ACT may appeal to some cultures in Africa. ACT belongs to the school of thought known as the third wave of behavioral and cognitive therapies (7, 8). ACT's fundamental principle boils down to “be present, open up and do what matters” (9) in order to lead a vital life in the presence of difficult thoughts and emotions.

The traditional CBT uses a content-driven approach, which aims to challenge distorted automatic thought contents that may predispose a person to mental illness (10, 11). ACT, on the other hand, does not primarily strive to fight the distorted thinking but rather aims to change one's relationship with distressing private experiences. ACT does not aim at changing the content of one's thoughts or emotions but rather weighs if the thoughts and emotions are consistent with one's valued living and committed action. If the thoughts and emotions are not consistent with the chosen life direction of the individual, then the person will not run away from the thoughts and emotions, but will treat them as background noise instead. ACT cultivates willingness to make room for difficult emotions in the service of pursuing valued living and engaging in committed actions. The primary goal of ACT is thus not to eliminate psychological symptoms, but rather to acquire the necessary skills to empower patients to pursue a vital and meaningful life in the presence of difficult and uncomfortable mental experiences (12).

Large and well-designed longitudinal studies show that psychological inflexibility, or rigidity, is associated with the development, chronicity, and complexity of mental and behavioral health problems (13, 14). Over 800 randomized trials have been conducted on ACT, finding it to be effective in managing a wide variety of health problems including anxiety, depression, substance abuse, diabetes mellitus, epilepsy (15) and other chronic health conditions (16–19), which is comparable to traditional forms of CBT (20). The goodness of fit with ACT has also been demonstrated in low- and middle-income countries (LMICs) (21).

### Six Core Processes of ACT

Psychological flexibility is brought about by employing six interrelated core processes (22): (1) Willingness: Also known as acceptance, willingness refers to actively remaining in contact with present inner experiences in the service of pursuing one's chosen values (12). The willingness to make room for uncomfortable emotions in the service of pursuing valued living is one of the key components of ACT. (2) Cognitive defusion is an antidote to fusion: The term cognitive fusion refers to what happens when one becomes overly attached to distressing inner experiences, leading to a problematic grip on behavior. For example, a cognitive fusion with the past includes rumination and regrets about past events, fusion with rules can be “I must not make a mistake.” Cognitive fusion sabotages one's ability to take actions in the direction of one's valued living. The problematic grip of cognitive fusion on behavior is challenged by the construct of cognitive defusion. For example, in a therapy session, the therapist may ask: “If you let the thought dominate your behavior (fusion), does it take you toward the life you want to live or away from the life you want to live (workability)?” (12). (3) Contact with the present moment: An important aspect of psychological flexibility is flexible attention to the present moment, which allows an individual to be fully engaged in the present moment in a voluntary, focused, and flexible way (12). It is an important skill because it expands the present moment at the expense of contracting regrets about the past and anxious preoccupation of the future (12). (4) Perspective taking: Also referred to as self-as-context, perspective taking refers to a person's attribute of observing one's actions, thoughts, and emotions. It is one's inherent attribute of being aware of one's awareness. This observing self is in contrast with self-as-content where one is fused with one's own story (12). (5) Value clarification: Identifying personal values is extremely important in ACT because they create a sense of purpose and meaning in life (12). Without identified values, one becomes radarless. (6) Committed action: ACT is, in essence, a behavioral intervention. This is embodied in committed action, which refers to directing one's behaviors to be consistent with one's values (12). The six ACT processes, humorously referred to as “hexaflex,” are interrelated. For example, when one takes actions, invariably, difficult and distracting emotions and thoughts show up. One must make room for these emotions in order to pursue committed action. Perhaps, this is best captured by a quote attributed to Elbert Hubbard: “Self-discipline is the ability to make yourself do what you should do when you should do it, whether you feel like it or not.”

### Proposed Conceptual Model

**Figure 1:**
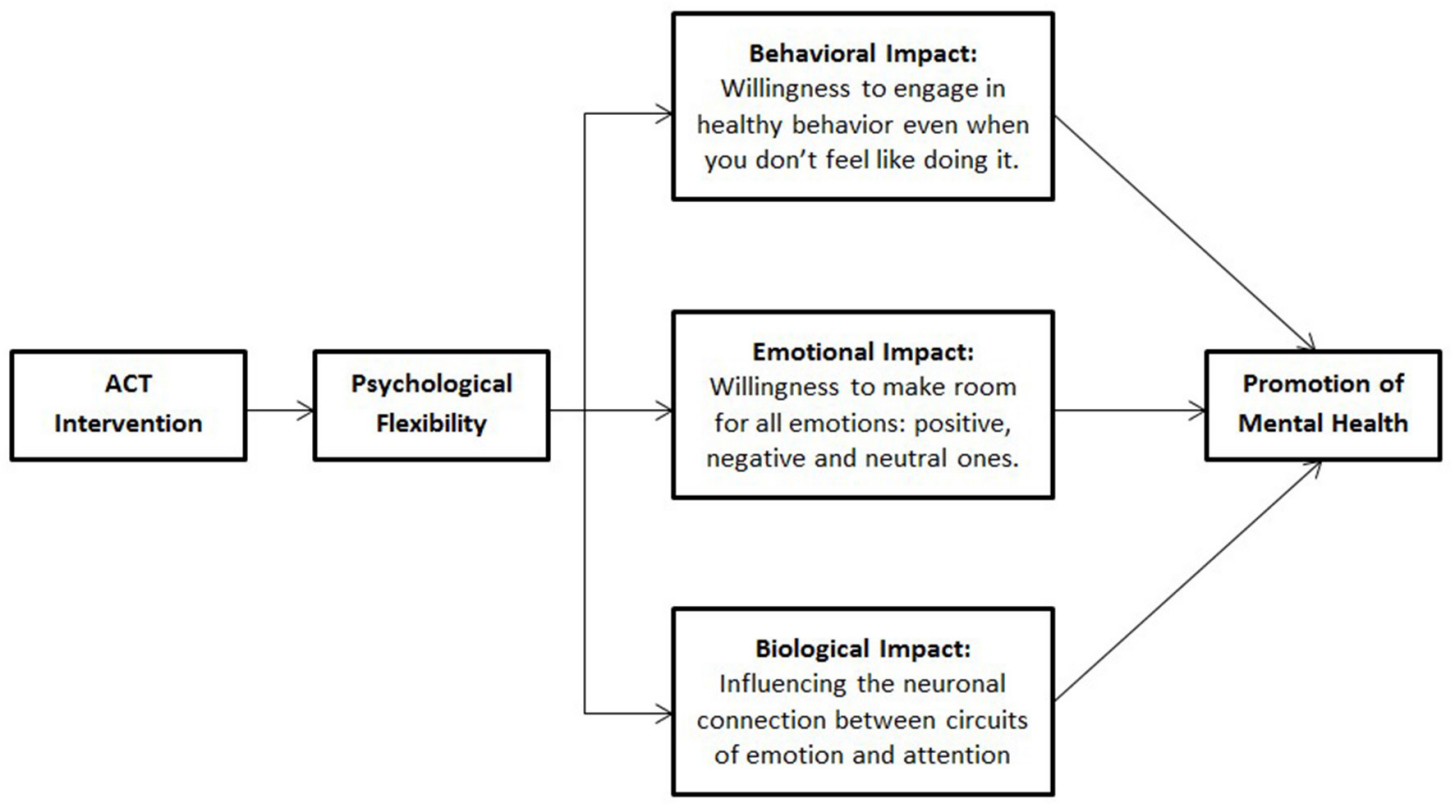
Proposed conceptual model.

Psychological flexibility is a mediator of mental health promotion in ACT (23). Here, we propose a model (Figure 1) that postulates four possible mechanisms through which ACT may promote mental health: (1) Increasing the ability of the individual to engage in healthy behavior even when one does not feel like doing it. However, the next key question is as to how one can do what he or she is supposed to do. This is achieved by cultivating the construct of willingness, i.e., to make room for uncomfortable emotions and thoughts that invariably show up when one is taking tangible actions in the direction of one's chosen values. The terms acceptance and willingness can be used interchangeably (24). This is primarily achieved through the combination of the psychological flexibility processes of values clarification and committed action, the latter of which may involve engaging in actions however imperfect they are (12). (2) Increasing emotional and cognitive flexibility. This is done by nurturing the person's willingness to experience all types of emotions, embodied by the process of acceptance. This will keep the distressing cognitions from halting action, which may lead to mental health promotion. (3) Increasing mindfulness skills: Allowing the person to be engaged in the present moment and focus attention voluntarily. This skill increases psychological flexibility and entails an insight about coming to terms with one's current circumstances, which in turn may promote mental health. This is captured in a quote attributed to Alfred D'Souza, “For a long time it had seemed to me that life was about to begin—real life. But there was always some obstacle in the way, something to be gotten through first, some unfinished business, time still to be served, or a debt to be paid. Then life would begin. At last, it dawned on me that these obstacles were my life.” (4) Psychological flexibility may also promote mental health by having a biological impact. Indeed, various randomized controlled studies have evaluated the effect of ACT-based interventions on brain processing using fMRI (25–28). For example, investigators from the University of Texas reported that an ACT-based intervention altered the default mode network, and reduced neurobiological activation during pain in the inferior parietal lobule, insula, anterior cingulate cortex, posterior cingulate cortex, and superior temporal gyrus among study participants with comorbid chronic pain and opioid addiction (25). Similarly, two studies from UCLA among individuals with social anxiety disorder showed an enhanced neural activity in the posterior insula (26) as well as changes in the amygdala-prefrontal functional connectivity (27) in participants that had been randomly assigned to an ACT intervention. Furthermore, a study conducted at Harvard University revealed changes in the ventrolateral prefrontal/ lateral orbitofrontal cortex in patients with fibromyalgia that underwent a 12-week ACT intervention (28).

It is important to note, that the data on ACT extends beyond affluent countries since more than 200 randomized controlled trials of ACT have been conducted in LMICs (29), and many of these have documented a significantly greater improvement in flexibility processes in ACT, even as compared to other evidence-based psychological interventions [e.g., (30)].

Few studies have shown that psychological flexibility is associated with mental health outcomes in an African context. For example, it mediates the positive impact of mindfulness on the distress of survivors of terrorist attacks (31), or the role of anxiety sensitivity on the distress of cardiac patients (32). Psychological flexibility also mediates the mental health outcomes of torture among refugees from sub-Saharan Africa (33). A systematic review of mediational findings of ACT randomized trials also provides support for the functionally important role of psychological flexibility in obtaining positive mental health outcomes (34). Nevertheless, there are not yet enough studies that examined the mediational role of psychological flexibility and/or the mental health impact of ACT interventions in Africa. This is also consistent with a recent publication in Lancet that highlighted the past and current mismatch in clinical trial efforts and disease burden globally, i.e., only 2% of randomized clinical trials registered between 2010 and 2019 were conducted in sub-Saharan Africa albeit this region is expected to have the most rapid population growth worldwide over the next decades (35).

## Mental Health Care Shortage in Africa—With a Focus on Ethiopia

Africa is the world's second largest continent in terms of both size and population. It is about 30.2 million square kilometers or 11.7 million square miles (about three times the size of the United States) with a population of about 1.2 billion people (36, 37). There are a total of 54 countries in Africa, most of which are considered LMICs. Recent literature has shown that LMICs in particular have a severe shortage of mental health services (38–41). The WHO estimates that there are 0.1 psychiatrists, 0.6 psychiatric nurses, and 0.1 psychologists per 100,000 people (42). These are staggeringly low ratios compared to the United States that has about 0.25 psychiatrists per 3,000 people as of 2017, and about 0.34 psychologists per 1,000 people as of 2014 (43, 44). Low numbers of medical graduates and outbreaks of diseases and infections pose additional barriers to effective health care in nations such as Ethiopia, where the majority of the population lives in remote, rural villages (45, 46).

Ethiopia, located in eastern Africa, is a LMIC with a major shortage of health services, including mental health services (47). Ethiopia is the second most populous country in Africa with over 110 million inhabitants and a land mass about twice the size of France (48). In 2016, Ethiopia reportedly had only about 40 psychiatrists and 15 psychologists (49) with current estimates of about 90 psychiatrists and 30 clinical psychologists. Up to 90% of people in need of mental health care may not get treatment due to lack of access (50). Over 80% of the population reside in remote, scattered rural areas, which makes it a huge challenge to provide consistent health care to all people (51–53). To address this problem, the Ethiopian Federal Ministry of Health (FMOH) launched a Health Extension Program (HEP) in 2004 (51). This program teaches Health Extension Workers (HEWs) various evidence-based interventions to address multiple health concerns under four major program areas: hygiene and environmental sanitation, family health services, disease prevention and control, and health education and communication (47, 51). For each village of about 5,000 inhabitants, two individuals, usually females, are recruited and trained in these essential health services for 1 year and then begin serving their community (54). As of 2013, more than 30,000 HEWs have been trained in these essential health services and deployed to about 15,000 villages in Ethiopia (51). However, as of today, the mental health package of HEWs in Ethiopia lacks an evidence-based psychological intervention that can be used by general health care workers. Without new approaches to expanding coverage to remote regions, increasing worker retention, and improving health outcomes, the situation will likely worsen.

One approach to overcome this limitation would be to train HEWs to deliver ACT under the supervision of mental health professionals. This would be a novel addition to the promotion of mental health and prevention of mental illness efforts across Ethiopia, including among individuals residing in rural areas. This goal could be achieved by implementing the well-established approach of task sharing (also referred to as task shifting) (55). With task sharing, non-specialist, general health care workers such as HEWs in Ethiopia can be trained in mental health care and psychological interventions that can then be provided to those in need (56). Indeed, ACT-based interventions have been delivered in different cultures using general health care workers (57, 58).

In addition, although there are minimal to no psychological services in Ethiopia, the culture itself provides a fertile ground to introduce a psychological intervention (59). For example, the transdiagnostic approach of ACT may appeal to Ethiopians whose traditional culture favors seeking counsel or advice in times of crisis from elders, neighbors, and friends (60). Indeed, Ethiopians generally rely on cultural resources for non-psychotic symptoms (50). ACT's emphasis on valued living and behavior may thus be compatible with the Ethiopian traditional culture of values.

To further address the question as to whether applying ACT may be a promising approach for tackling the mental health care shortage in Ethiopia, we also reviewed previous studies that have examined the effectiveness of ACT-based interventions in African countries.

## Overview: ACT-Based Interventions in Africa

To the best of our knowledge, only few studies exist that were conducted in Africa and investigated the effectiveness of an ACT-based intervention on outcomes related to mental health, and no study to date has been conducted in Ethiopia (Table 1).

**Table 1:** Overview of ACT-based intervention studies in Africa.

**References; Country**	**Study design**	**Study population**	**Intervention**	**Results**
Babalola and Ogunyemi (61); Nigeria	Pre-post randomized controlled trial	Adolescents in public secondary school in Nigeria with social phobia (*N* = 104)	Two groups: *ACT intervention* (*N = 53*): Weekly 30 min session for 8 weeks *Control group* (*N = 51*): Weekly leadership style teaching for 8 weeks	- Significant reduction of social phobia as measured by Social Phobia Inventory among secondary school adolescents with ACT intervention when compared to control group (*p* < 0.05)
Ishola et al. (62); Nigeria	Randomized controlled trial	HIV-positive pregnant women (*N* = 132; 31.6 ± 4.5 years; 91% married; 69% at least secondary education)	Randomly assigned to four groups (two control, two intervention groups): *Intervention groups*: HIV counseling + one session of ACT + weekly ACT text messages for 3 months *Control groups*: HIV counseling	- Increase in psychological flexibility as measured by Action and Acceptance Questionnaire from pre- to post-test (*p* = 0.023) in one intervention group, and significant decrease in one control group - Improvement in psychological flexibility between pre- and post-test in participants following ACT intervention as compared to control group (*p* = 0.001)
Lundgren et al. (15); South Africa	Randomized controlled trial	Adults with an EEG-verified epilepsy diagnosis with drug refractory seizures (*N* = 27; 14 females)	Randomly assigned to control or intervention group *Intervention group*: 1.5 h individual ACT session, two 3 h group ACT sessions, final 1.5 h individual ACT session over 5 weeks. Additional 1-week periods at 6 and 12 months. *Control group*: Supportive therapy	- Increase in life satisfaction as measured by Satisfaction with Life Scale (*p* < 0.05) in intervention group at every posttreatment comparison (post, 6-months post, 1 year-post) - Increase in quality of life as measured by WHO Quality of Life questionnaire (*p* < 0.05) in intervention group at 1 year posttreatment comparison - Decrease in seizure frequency and seizure index in intervention group at every posttreatment comparison (post, 6-months post, 1 year-post) between *p* < 0.048 and *p* < 0.011
Ochuba and Abamara (63); Nigeria	Pre-post within-subjects design	Patients diagnosed with substance use disorder and suffering from addiction to psychoactive drugs (*N* = 60; 50 males; 34 ± 7.93 years)	Three groups: *ACT intervention* (*N = 20*): 1 h individual session, twice per week for 10 weeks *Psychoeducation intervention* (*N = 20*): 1 h individual session, twice per week for 10 weeks *Control* (*N = 20*): No psychological intervention	- ACT intervention reduces symptoms of patients suffering addiction to psychoactive drugs when compared to psychoeducation intervention (*p* = 0.002) - Significantly lower symptoms shown with ACT intervention when compared to control group (*p* = 0.001) - Significantly lower symptoms shown with ACT intervention when compared to psychoeducation intervention (*p* = 0.007)
Oluwole (64); Nigeria	Randomized controlled trial	Spiritually abused senior secondary public school students in Nigeria (*N* = 180; 90 males; 15.6 ± 2.61 years)	Three groups: *ACT group* (*N = 60*): One 40-min ACT session per week for 8 weeks *DBT group* (*N = 60*): One 40-min DBT session per week for 8 weeks *Control group* (*N = 60*): Participants received no treatment	- Significant main effect of treatments on social competence (*p* < 0.05) as measured by Social Competence Rating Scale - ACT was more effective than DBT and control condition in enhancing social competence
Stewart et al. (58); Sierra Leone	Preliminary evaluation study	Non-specialist health workers and professionals (*N* = 57; 31 females; 34 ± 7.69 years)	Three-day ACT workshop comprised of didactic instruction led by facilitators with role plays in front of the group, then divided into small groups to practice	- Increase in psychological flexibility as measured by Acceptance and Action Questionnaire-II, from baseline to 3-months post-baseline (*p* = 0.001) and from post-workshop to 3-months post-baseline (*p* = 0.013) - Increase in life satisfaction as measured by Satisfaction with Life Scale, from baseline to 3-months post-baseline(*p* = 0.017) and from post-workshop to 3-months post-baseline (*p* = 0.037) - No significant effects for Valuing Questionnaire - Findings indicate acceptability of undergoing ACT training
Tol et al. (65); Uganda	Feasibility cluster randomized controlled trial	South Sudanese refugee women (*N* = 50; 29.5 ± 8.5 years; 68% married)	Two groups: *ACT-based Self-Help Plus* (*SH±*) *intervention* (*N = 25*): Intervention supervised by social worker and delivered by Ugandan women without prior mental health experience *Enhanced usual care* (*N = 25*): Comprised one psychoeducation session delivered by a trained community health worker	- Larger mean post-intervention differences for the SH+ condition on all outcome measures - Decrease in psychological distress as measured by Kessler 6 (*p* < 0.05) - Increase in subjective well-being as measured by WHO Wellbeing Index (*p* < 0.001) - SH+ research protocols deemed feasible
Tol et al. (66); Uganda	Cluster randomized controlled trial	South Sudanese refugee women with at least moderate levels of psychological distress (*N* = 694; 30.9 ± 10.9 years; 60% married)	*ACT-based Self-Help Plus* (*SH±*) *intervention* (*N = 331*): Access to usual care and five 2-h audio-recorded stress-management workshops led by trained lay facilitators, accompanied by an illustrated self-help book *Enhanced usual care* (*N = 363*): Comprised one psychoeducation session delivered by a trained community health worker	- Stronger improvements for SH+ on psychological distress as measured by Kessler 6 immediately after intervention (*p* < 0.001) and at 3 months post intervention (*p* = 0.04) - Larger improvements for SH+ at 3 months post-intervention for five of eight secondary outcomes (post-traumatic stress, depression symptoms, explosive anger, functional impairment, subjective well-being) - Refugees with different trauma exposure, length of time in settlements, and initial psychological distress benefited similarly

For example, one study from Sierra Leone examined the acceptability and mental health impact of a 3-day ACT training workshop for 57 non-specialist health workers and professionals. The investigators reported an improvement in psychological flexibility and life satisfaction both post-workshop as well as after a 3-month follow-up (58).

Additionally, six studies investigated the impact of an ACT intervention on mental health outcomes in different groups of participants, i.e., South Sudanese refugee women in Uganda (65, 66), HIV-positive pregnant women in Nigeria (62), adults with epilepsy in South Africa (15), spiritually abused senior secondary public school students in Nigeria (64), adolescents with social phobia attending public secondary school in Nigeria (61), and patients diagnosed with substance use disorder and suffering from addiction to psychoactive drugs in Nigeria (63). In a feasibility study from Uganda published in 2018 (65), 25 South Sudanese refugee women underwent an ACT-based stress management intervention called Self-Help Plus (SH+), consisting of two components: a pre-recorded course and a self-help book (67). The intervention was delivered by Ugandan women without prior mental health experience that were trained to administer the intervention and supervised by a social worker (65). The same group of investigators 2 years later published a randomized controlled trial including a much larger sample of 694 South Sudanese refugee women and the same study methodology, i.e., 331 participants received the SH+-intervention and 363 participants received enhanced usual care (66). Another study took place in Nigeria where 132 pregnant HIV-positive women were randomly assigned to either a control or intervention group. The intervention group received post-HIV test counseling and one session of ACT followed by weekly text messages based on ACT for 3 months; the control group received only the counseling (62). In the South African study, 27 adults with an EEG-verified epilepsy diagnosis with drug refractory seizures were randomly assigned to either a control group (supportive therapy) or an ACT intervention group. The ACT intervention consisted of a 1.5-h individual session, followed by two 3-h group sessions, and a final 1.5-h individual session over 5 weeks. After 6 and 12 months, participants received another 1 week of intervention (15). Finally, three studies were conducted in Nigeria. One RCT included 180 spiritually abused students and found that a weekly 40-min ACT session delivered over 8 weeks was more effective than a DBT intervention and control condition in increasing social competence (64). Another RCT among 104 adolescents with social phobia compared an 8-weeks ACT intervention to a control condition, and researchers observed a significant reduction of social phobia in the ACT intervention group (61). The third study from Nigeria recruited 60 participants diagnosed with substance use disorder and suffering from addiction to psychoactive drugs and randomly assigned them to either an ACT intervention group, psychoeducation intervention group or control group. Again, the ACT intervention was most effective in reducing symptoms of those participants (63).

Though these studies differed in terms of study population, characteristics of the ACT intervention and measurements, all revealed significant improvements of various mental health-related outcome measures in the groups that received an ACT-based intervention, such as decreased psychological distress and increased subjective wellbeing (65, 66), improved post-traumatic stress, depression symptoms, explosive anger, and functional impairment (66), increased psychological flexibility (58, 62), increased life satisfaction (58), increased social competence (64), reduced social phobia (61), and reduced symptoms of suffering from addiction to psychoactive drugs (63). Of note, ACT was also associated with significant decreases in seizure frequency and seizure index among epilepsy patients but not in the supportive therapy groups (15).

Only two of the studies we identified have used a task sharing approach similar to what has been proposed by the WHO, i.e., only the studies from Uganda trained individuals without prior mental health experience to deliver an ACT intervention (65, 66). In addition, the investigators from Sierra Leone have examined the acceptability of completing an ACT workshop among non-specialist health workers and also tested whether the training is associated with psychological flexibility and life satisfaction in these trained individuals. Furthermore, ACT research using a “train-the-trainer” approach is currently being carried out in Sierra Leone, where couples are taught partnering skills and offered resources for teaching these skills to other couples, but results have not been published yet.^1^^,^^2^

Overall, given the small number of studies that administered an ACT intervention in African countries, more research is needed to investigate the feasibility and acceptability of training non-specialist health care workers in administering ACT-based interventions in African countries, particularly LMICs, with severe shortage of mental health services such as Ethiopia. It is paramount to examine whether ACT-based interventions delivered by trained health care workers have an impact on mental health outcomes (both in the short- and long-term) in various groups of individuals including, but not limited to, persons with specific mental and behavioral health problems, such as depression or anxiety, as well as transdiagnostic conditions, such as perceived stress or chronic diseases. To our knowledge, there is currently no study that investigated the effect of an ACT-based intervention on mental health outcomes in Ethiopia. As mentioned above, Ethiopia is the second most populous country in Africa, and additionally houses the headquarters of African Union, thus creating a logistic opportunity to scale up evidence-based psychological interventions *via* the education commission of the African Union based in Addis Ababa, Ethiopia. This review paper authored by a group of investigators from the US, Germany, and Ethiopia, paves the way for a planned ACT intervention to be delivered by laypersons, i.e., HEWs, and supervised by ACT-trained mental health professionals in Ethiopia.

## Evidence for ACT Delivered by Non-Specialists

To date, at least nine RCTs have evaluated ACT-based interventions that were self-administered or delivered by non-specialists outside of the African context. One study from Sweden compared ACT self-help to moderated online discussion forums for chronic pain patients with functional impairments and found significant improvements in acceptance and activity engagement as well as reductions in depression, stress, and anxiety that maintained at 6-month follow-up in the ACT group (68). Another Swedish research found strong adherence to a behavioral activation with ACT self-help protocol for patients with depression and previous histories of dropping out of clinical programs. At posttreatment, 25% of the treated group reached remission criteria compared to only 5% of the controls (69). A RCT from the Netherlands compared ACT self-help with minimal and extended support, finding that those with more email interactions with supporters showed stronger acceptance outcomes and significant reductions in depression and anxiety (70). It is important to note that participants in this study showed mild to moderate depressive symptomology and were recruited from the general population. Thus, this study lends support to the notion that a population-level application of ACT can be efficacious. Swedish investigators found that internet-delivered ACT was equivalent to internet-based CBT for participants with tinnitus distress (71). Jeffcoat and Hayes (72) offered an ACT self-help book to teachers and reported that teachers read the book, completed exercises, and showed strong acceptance outcomes. The intervention was associated with preventive effects for depression and anxiety among those with no symptomology at the onset of treatment and significant ameliorative effects for those in the clinical ranges of depression, anxiety, and stress. Similarly, researchers from New Zealand gave an ACT self-help book to individuals experiencing chronic pain and offered weekly telephone support from a non-professional; the investigators found that using the self-help book, with some telephone assistance, improved quality of life and decreased anxiety in participants (73). Investigators from Finland combined CBT and ACT self-help intervention with personal health technology that included online group meetings, an internet portal, mobile phone applications, and personal monitoring devices for men with sleep disturbances (74). They observed reduced depressive symptomology as well as increased health and working ability in the treatment group, and 10 out of 11 participants used at least one of the assistive technologies. Importantly, participants with diagnoses were not included in the study and thus the group meetings were not considered psychotherapy sessions. In another study, Japanese college students living in the United States were given an ACT self-help book and the investigators found that moderately depressed or stressed and severely anxious students showed improvements compared to those that did not receive the book; and when the wait list participants were given the book, they showed equivalent results (75). Finally, Swedish investigators compared ACT self-help to relaxation self-help interventions and reported that ACT significantly increased acceptance of chronic pain, improved life satisfaction, and led to higher levels of functioning in the presence of pain, compared to applied relaxation. The two treatments had equivalent effects upon depression and anxiety (76).

Taken together, these RCTs show that ACT can be delivered by non-specialists and that individuals with minimal literacy skills can make substantial improvements with self-help training materials. Four of the nine studies reviewed involved self-help materials presented and coached by a non-specialist. Combined with the studies reviewed in the previous sections, there is reason to believe that HEWs can be counted on to deliver ACT or support individuals learning to use ACT from self-help materials across rural Ethiopia. This is important because although studies conducted in the West show that participants respond to ACT delivered in the form of self-help products, in Ethiopia and other African nations, someone would be needed to deliver the materials, introduce them to participants, and offer support to those with more minimal literacy skills. Of additional note, the conceptual model we described above is consistent with this literature. The trend suggests that ACT interventions can lead to improved mental health outcomes that translate into increased adherence with public health guidelines, vaccination schedules, doctor visits, and physician recommendations.

## Recommendations for Additional Empirical Support

Nevertheless, given the small number of studies conducted in sub-Saharan Africa showing the effects of ACT delivered absent the presence of professional health care workers, we recommend the following efforts to generate additional process and outcome data:

First, studies are needed to lend support to the conceptual model described herein. We have proposed that ACT interventions result in psychological flexibility characterized by changes in overt behavior, emotional acceptance, and neuronal within- and between- brain region connectedness. Additional studies are needed to evaluate (a) the strength and durability of behavioral changes at different doses of ACT delivered by non-specialists, (b) the extent of willingness participants develop for witnessing their own suffering as they engage in life-enhancing, healthy behaviors, and (c) the degree to which biological changes participate in values-based behavior and emotional engagement.

Second, extensive research is needed on task sharing and the use of HEWs in rural regions of Ethiopia and other nations in sub-Saharan Africa. To date, only a handful of studies show that task sharing is efficacious, despite the embrace of this strategy by the WHO. The extent of training for support personnel that is needed as well as the degree to which telephones, internet, and email correspondence can be relied upon to augment mental health care services in Ethiopia and other LMICs are in need of further investigation. In addition, although some studies have used a train-the-trainer approach, more studies are needed to evaluate whether these strategies can be used in remote regions with limited direct contact with researchers and clinicians.

Third, only a few studies have applied ACT in African nations. More such studies are needed to parse those components of the psychological flexibility model most needed to transform the aversive functions of behavior, thoughts, emotions, and stimuli associated with mental health. We have described a framework that includes emotional, attentional, cognitive, and perspectival process components necessary for evoking values-based behavior. However, it is possible that more attention should be paid to motivational and behavioral components of the ACT model, and less to the four we have focused on. Studies are needed to determine the components and dosages needed in the design of a population-level ACT intervention.

Fourth, as intimated in the first recommendation, researchers should investigate the degree to which self-help, non-specialist coaches, and HEWs are needed. It may be that a tiered model incorporating all of these is needed; alternatively, one of these or a combination of two could provide the necessary and sufficient conditions for promoting behavior that fosters mental health in Ethiopia and other sub-Saharan LMICs.

## Conclusions

Improving the access to and outcomes of mental health care in Africa is crucial to facilitate disparities reduction. A sustainable, evidence-based mental health intervention in Africa should address several challenges and barriers, e.g., incorporate the needs and expectations of the local population, be consistent with cultural belief systems, facilitate interprofessional communication, or incorporate a public health approach (77). A Cochrane review (78) found that health workers can produce beneficial outcomes in LMICs, but only a handful of studies used evidence-based psychosocial interventions, and only one did so in Africa, testing the impact of group interpersonal psychotherapy in Uganda (79). Consistent with the WHO recommended approach of task sharing for mental health care in LMICs, ACT may be delivered by trained laypersons who have a grassroots presence. To date, only few studies have investigated the effectiveness of an ACT-based intervention in African countries including Uganda, Sierra Leone, Nigeria and South Africa, and these preliminary data have shown promise for the effectiveness of ACT in reducing the mental health care disparities. The regional stakeholders that may be interested in research on ACT and the delivery of an ACT-based intervention delivered by laypersons include the Ethiopian Ministry of Health that has successfully trained HEWs to implement an exemplary HEP (54, 80, 81). Ethiopia has earned international reputation for establishing a network of HEWs in order to promote health and prevent diseases. This infrastructure would provide a solid basis for introducing, testing and, if proven successful, scaling up a task sharing approach of an ACT-based intervention being delivered by trained HEWs at a grassroots level, possibly paving the way for an innovative, sustainable mental health service in Ethiopia, and other African countries.

## Data Availability Statement

The original contributions presented in the study are included in the article/supplementary material, further inquiries can be directed to the corresponding author/s.

## Author Contributions

YG, JK-R, and YF drafted the manuscript. TS and SH wrote sections of the manuscript. AT, TB, DR, and MA provided critical revision of the manuscript. All authors have read and approved the final manuscript.

## Funding

The authors wish to acknowledge the Edli Foundation (Netherlands) for providing funding. This work was in part funded by the National Institutes of Health (R01AG057708).

## Conflict of Interest

The authors declare that the research was conducted in the absence of any commercial or financial relationships that could be construed as a potential conflict of interest.

## Publisher's Note

All claims expressed in this article are solely those of the authors and do not necessarily represent those of their affiliated organizations, or those of the publisher, the editors and the reviewers. Any product that may be evaluated in this article, or claim that may be made by its manufacturer, is not guaranteed or endorsed by the publisher.

## Footnotes

^1^Szabo TG, Bockarie H. ACT training for Sierra Leonean Couples: a train-the-trainer model. Personal communication (2020).

^2^Szabo TG, Bockarie H, White R, Tarbox J, Stewart C, Ebert B. Microaggression, intimate partner gender-based violence, and behavioral flexibility training in Sierra Leonean couples. Personal communication (2020).
